# A measured energy use, solar production, and building air leakage dataset for a zero energy commercial building

**DOI:** 10.1038/s41597-021-01082-8

**Published:** 2021-11-23

**Authors:** Philip Agee, Leila Nikdel, Sydney Roberts

**Affiliations:** 1 Myers-Lawson School of Construction, Virginia Tech, Blacksburg, VA 24061 United States; 2 Apogee Interactive, Tucker, GA 30084 United States

**Keywords:** Energy efficiency, Energy and society

## Abstract

This paper provides an open dataset of measured energy use, solar energy production, and building air leakage data from a 328 m^2^ (3,531 ft^2^) all-electric, zero energy commercial building in Virginia, USA. Over two years of energy use data were collected at 1-hour intervals using circuit-level energy monitors. Over six years of solar energy production data were measured at 1-hour resolution by 56 microinverters (presented as daily and monthly data in this dataset). The building air leakage data was measured post-construction per ASTM-E779 Standard Test Method for Determining Air Leakage Rate by Fan Pressurization and the United States Army Corps (USACE) Building Enclosure Testing procedure; both pressurization and depressurization results are provided. The architectural and engineering (AE) documents are provided to aid researchers and practitioners in reliable modeling of building performance. The paper describes the data collection methods, cleaning, and convergence with weather data. This dataset can be employed to predict, benchmark, and calibrate operational outcomes in zero energy commercial buildings.

## Abstract (summary)

**Table:** 

Measurement(s)	measured energy use • solar energy production • building air leakage
Technology Type(s)	circuit-level energy monitor • microinverter • ASTM-E779 Standard Test Method for Determining Air Leakage Rate by Fan Pressurization
Factor Type(s)	temporal interval
Sample Characteristic - Environment	zero energy commercial building
Sample Characteristic - Location	Commonwealth of Virginia

Machine-accessible metadata file describing the reported data: 10.6084/m9.figshare.16869407

## Background & Summary

Significant data is generated in the built environment. Design and construction documents, performance simulations, commissioning results, and building performance data (BPD) are generated on all modern buildings. Today, system advancements in energy efficiency, integration, monitoring, and connectivity are expanding our design and facility management options throughout the built environment. Internet-enabled monitoring, ubiquitous user interfaces, machine learning, and artificial intelligence are facilitating analytical approaches not previously afforded to our industry. As buildings become more complex, the need to measure, analyze, and share BPD has become salient.

Open datasets are critical for advancing building performance and analytical methods. Open BPD can be leveraged to calibrate design simulations and inform future design, construction, and operational decisions. Open BPD can facilitate benchmarking performance across building types, vintage, and geographic distributions^1^. Conversely, open BPD can be used to benchmark machine learning techniques for the built environment^2^. Simply put, open BPD is a critical component in the systems approach necessary to improve outcomes in the built environment including, but not limited to reducing carbon emissions and operating costs and improving human experiences in the built environment.

The Architecture, Engineering, Construction, and Operation (AECO) industry rarely systematically collects or shares BPD. Researchers increasingly collect BPD, yet rarely share BPD in open-source data repositories^3^. In recent years, however, there has been a trend to develop and share open-source BPD^4–12^. For example, there are recent open BPD contributions focused on occupant behavior impacts on energy use and indoor environmental quality (IEQ).

These contributions span human-building interactions with appliances^5^, heat pumps^7^, and natural ventilation systems^8^. Other recent contributions span energy use across building typologies (e.g., commercial and residential)^9–12^. This paper contributes to the recent trend toward open BPD datasets.

There are two novel contributions of this work. First, the longitudinal energy use and energy production data can be employed in the pursuit of zero energy building design and performance. As we race toward zero energy buildings, open BPD become critical for reducing performance gaps between expected and realized performance. The volume and variety of small commercial buildings compounds the need to go beyond simple benchmarking exercises and requires higher resolutions of data to achieve performance targets. The second contribution of this work is the building air leakage dataset combined with longitudinal energy use and energy production data. While building air leakage is an important factor in predicting energy use, estimating heating and cooling loads, and designing for indoor environmental quality (IEQ), it is rarely available in open BPD. The balance of this paper characterizes the case study project, data collection methods, and resulting data records.

## Methods

### Case study context

This dataset was developed from a single, non-random case study project. The building serves as a leasing office and community building for a national non-profit housing provider (referred hereafter as the “owner”). The owner’s mission is to create homes and communities that are healthy, sustainable, and affordable. The building was designed in 2013 and construction was completed in April 2014. The owner pursued EarthCraft Light Commercial (ECLC), a regional 3rd party green building program. The ECLC program was used to verify high performance design and construction targets were achieved. Table 1 provides an overview of the building specifications and the following section characterizes the data collection techniques for the energy use (demand over time – kWh), energy production, and building air leakage data.

**Table 1:** Building specifications.

Category	Parameters	Building Characteristics
General	Climate zone	Mixed-humid, US 4 A
	Building type	Office
	Area	328 m^2^ (3,531 ft^2^)
	Schedule	1st FLR 08:30–15:00
		2nd FLR 08:30–17:00
	Occupancy	1st FLR: n = 3
		2nd FLR n = 3–20 depending on classes
Enclosure	Enclosure surface area	1,155 m^2^ (12,437 ft^2^)
	Window U-value	0.30 BTUh/ft^2^/°F (1.70/W/m²K)
	SHGC (g-value)	0.22
	Visible light transmittance	0.41
	Wall U-value	0.04 BTUh/ft^2^/°F (0.13 W/m²K)
	Roof/Attic U-value	0.02 BTUh/ft^2^/°F (0.11 W/m²K)
	Air tightness	1.02 L/s-m^2^ at 75 Pa (0.20 cfm_75_/ft^2^)
Systems	Heating system	Air-source heat pump, 9 HSPF (n = 3)
	Cooling system	Air-source heat pump, 15.5 SEER (n = 3)
	Distribution	3 ducted air systems, within thermal boundary
	Temperature control	Programmable thermostat (n = 3)
	Water heating	30-gallon, electric storage (0.93 EF)
	Ventilation	Energy recovery ventilator
	Int. Lighting Power Density	7.62 W/m^2^ (designed)
	Ext. Lighting Power Density	1.21 W/m^2^ (designed)
	Solar PV-system	75.2 m², 12.3 kWp, panels & microinverters (n = 56), Azimuth: 135°

### Energy use

Energy use data (demand over time - kWh) were measured using a SiteSage energy monitoring system. 150 A current transformers (CTs) were installed on the building electrical mains, while 20 A or 50 A CTs were installed on the balance of the building’s circuits depending on the circuit load (e.g., water heating, lights, heat pump). Thirty discrete circuits are measured and reported in the dataset. Each CT transmitted energy use in one-hour intervals to an internet-enabled gateway located in the electrical panel. Data was accessed via an online user interface and downloaded as a .csv file for the purposes of this data descriptor. It is important to note that for safety reasons related to accessing a live electrical panel, a licensed electrician installed the CTs in the presence of the corresponding author.

### Energy production

**Fig. 1:**
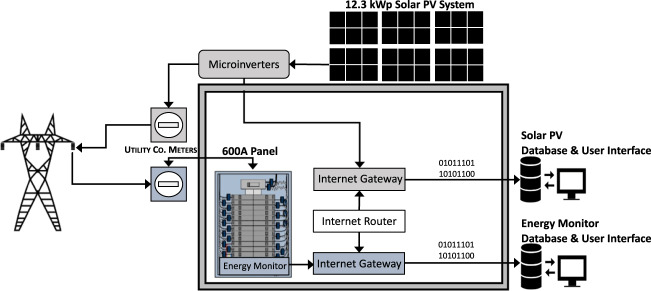
Sensor architecture.

Energy production data were measured from the building’s roof mounted, 12.3 kWp solar photovoltaic (PV) system. The solar PV system was equipped with 56 microinverters that reported production data via power line communications to an Enphase internet-enabled gateway. The microinverter production data is aggregated and reported in daily intervals. Data was accessed through an online user interface and downloaded as .csv files for the purposes of this data descriptor. The energy production monitoring system was installed by a licensed solar contractor. Access to the user interface and data was provided to the authors by the owner. Figure 1 details the sensor architecture for the energy use and energy production data collection.

### Building air leakage

The case study’s building enclosure was tested at the end of the project’s construction phase in April 2014. Building air leakage data were measured per the U.S. Army Corps of Engineers (USACE) Air Leakage Protocol for Building Envelopes^13^ and ASTM E779 - Standard Test Method for Determining Air Leakage Rate by Fan Pressurization^14^. Building air leakage rates were measured with two calibrated blower door fans, and two calibrated digital micromanometer gauges. Category 5 (CAT5) cable was used to connect the gauges to TECLOG3, a data logging software developed by the fan and gauge manufacturer. The software was also used to provide results in compliance with ASTM E779 (described in the Data Records section of this descriptor).

**Fig. 2:**
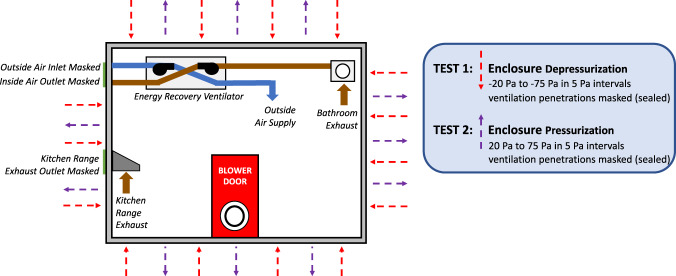
USACE Building Enclosure Testing procedure.

The USACE protocol requires two tests: a multi-point depressurization test and a multi-point pressurization test. During the test, all intentional enclosure penetrations (e.g., outdoor air, make-up air, and exhaust air penetrations) are sealed with masking. Following a pre-test baseline, ten points were collected from ±20 Pa (0.08 inH_2_O) to ±75 Pa (0.3 inH_2_O) at 5 Pa (0.02 inH_2_O) intervals. Following each test, a post-test baseline was measured. Each point was averaged over 10 seconds. Figure 2 provides a representation of the building air leakage tests per the USACE protocol.

## Data Records

The primary and secondary data that accompany this data descriptor are provided at the *Open Science Framework* (OSF) respiratory^15^. The dataset is organized in five categories: (1) measured energy use, (2) measured energy production, (3) measured building air leakage, (4) weather, and (5) architectural and engineering (AE) documents. All data is provided in .xlsx, with the exception of the EnergyPlus weather files and AE documents. Table 2 characterizes the study’s primary data records.

**Table 2:** Primary data output overview.

Category	Data Description	Unit	Data Resolution	Data Output
Energy Use	Circuit-level energy consumption	kWh	• Hourly	• HVAC.xlsx
			• Daily	• Lighting.xlsx
			• Monthly	• Plug & Process Loads.xlsx
				• Whole Building.xlsx
Energy Production	Solar energy production	Wh		• Energy Production.xlsx
		kWh	• Daily	
			• Monthly	
Building Air Leakage	Mean building pressure	Pa	Discrete test (n = 2)	• Building Air Tightness.xlsx
	Mean building air flow	CFM_75_		
	Mean building pressure	(−)Pa		
	Mean building air flow	CFM_−75_		

To assist users of this dataset, the authors provide AE documents, publicly available weather data, and the EnergyPlus weather file (.EPW) for the case study building’s site. These data are considered as secondary data since they were not directly measured by authors. Table 3 provides an overview of the secondary data for the period of data measurements.

**Table 3:** Secondary data overview.

Category	Data Description	Unit	Data Resolution	Data Output
Weather	Temperature	°C (°F)	Daily	• Weather_Daily_IP.xlsx
			Monthly	• Weather_Monthly_IP.xlsx
				• Weather_Monthly_SI.xlsx
	Dew point	°C (°F)	Daily	
			Monthly	
	Humidity	%	Daily	
			Monthly	
	Wind speed	m/s (mph)	Daily	
			Monthly	
	Pressure	kPa (Hg)	Daily	
			Monthly	
	Precipitation	cm (inch)	Daily	
			Monthly	
	Heating degree days	# of days	Monthly	
	Cooling degree days	# of days	Monthly	
	Solar radiation	W/m^2^	Hourly	• Solar Radiation_Daily.xlsx
			Daily	• Solar Radiation_Hourly.xlsx
			Monthly	• Solar Radiation_Monthly.xlsx
	EPW, DDY, and STAT	N/A	N/A	• AP.724016_TMY3.zip
AE Documents	Architectural, mechanical, electrical, plumping plans, solar PV plans	N/A	N/A	• AE Documents.pdf
				• Solar PV System Schematic Plan.pdf

### Energy consumption

Circuit-level energy consumption data (demand over time – kWh) were measured from June 18, 2014 to December 13, 2016. Data is organized into four datasets presenting energy use for the whole building and three end-use categories: lighting, plug & process loads (PPLs), HVAC & hot water energy use. Each dataset has circuit-level hourly data and aggregated data for total consumption for the whole building at hourly, daily, and monthly resolutions presented at separate tabs. In addition to the total energy use for the whole building and the three end-uses, energy use for first floor vs. second floor, interior vs. exterior lights, and HVAC vs. hot water are provided in the end-uses datasets in the tabs for hourly, daily, and monthly aggregated data.

Monthly energy use for the whole building varies from 985 kWh to 2,482 kWh over the measurement period (with the monthly average of 1,499 kWh). Average daily energy use over the whole measurement period is 49.4 kWh per day. Higher energy use for HVAC, hot water, and interior lighting is observed in colder months with longer night hours. Seasonal variations for HVAC energy use are over 1,000 kWh while interior lighting is about 100 kWh. No major seasonal or monthly variations were observed for exterior lightings. However, there is an increase in energy use for exterior lighting from December 2014 due to a change in the control system that resulted in some of the lights being on consistently during the day. Monthly energy use for PPLs were below 300 kWh per month with a fluctuation of 100 kWh between different months from July 2014 to October 2015. No major outliers were found in the datasets. A few outliers in PPLs were observed likely due to human-building interactions. Data for energy recovery ventilation (ERV) systems are included in the dataset, but it should be noted that the ERV was unplugged by the owner within the first month of operation due to concerns related to indoor humidity.

### Energy production

Energy production data for the period of May 15, 2014 to December 31, 2020 is provided in a single .xlsx file. Data is organized in daily and monthly resolutions presented at separate tabs. Although data was measured at 1-hour intervals, the hourly resolution of data is not included in this dataset as the data export function on the solar PV user interface only exports measured data in daily intervals. Daily data was aggregated into monthly data in Excel and examined for missing data. Years 2015, 2016, and 2017 have missing data for two to eight months in the year due to accidental system shutoffs. Overall, 66 months have complete data for all days in the month. Missing data were shown in grey cells in the both daily and monthly data tabs. Monthly averages over the whole 7-year period and a 3-year period with complete data for all months of the year (2018 to 2020) are also provided in the monthly data tab. Minimum, maximum, and average values for daily energy production are provided in the daily data tab.

**Fig. 3:**
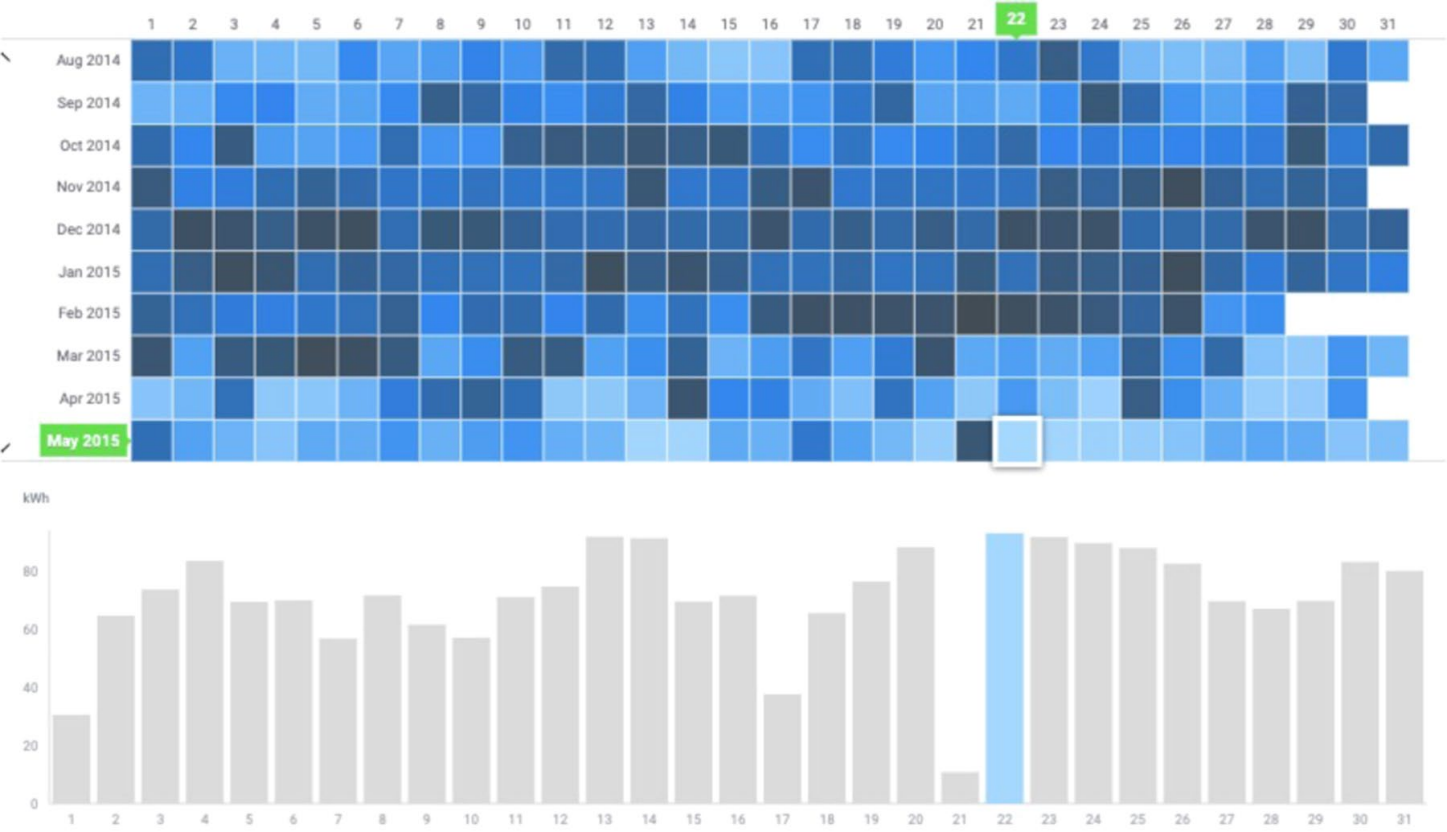
Example of daily energy production data. Lighter shades of represent higher energy production.

The Annual average of energy production is 1,218 kWh for years without missing data (2018 to 2020). More than 1,000 kWh energy is produced in a month from March through October. More energy production, however, was observed in April to August with July having the highest energy production on average over 7 years of data measurement. The highest monthly energy production was recorded as 2,213 kWh in May 2015. Figure 3 presents variations in daily energy production from August 2014 to May 2015.

### Building air leakage

Building air leakage data is provided in a single .xlsx file. The USACE protocol and ASTM E779 standard require the reporting of both depressurization and pressurization tests. Results are separated by tab in the file. Each test (e.g., tab) reports three columns of data: (1) test point, (2) average induced enclosure pressure (+/− 75 Pa), and 3) total corrected fan flow (CFM).

### Weather data

An EnergyPlus weather file (.EPW) for Charlottesville, VA is provided for building energy simulation applications^16^. Measured weather data for Charlottesville, VA provided by Weather Underground website^17^ from June 2014 to December 2016 is also available in the OSF respiratory. Daily and monthly resolutions of weather data are provided into two separate subfolders in the respiratory. Weather data includes temperature, dew point, humidity, wind speed, pressure, and precipitation. Daily weather data in the .xlsx file is provided in separate tabs for each month of the energy use measurement period. The average for each month is presented in the last row in the tab for that month. Monthly weather data includes heating degree days (HDDs) and cooling degree days (CDDs) and is provided in both SI and IP unit systems. The base temperature was 18 °C (65 °F) for both HDDs and CDDs. To facilitate investigating of the correlation between solar radiation and solar energy production, solar radiation data is provided from the National Oceanic and Atmospheric Administration (NOAA) website^18^ for Charlottesville, VA for the energy production measurement period (2014 to 2020) in hourly, daily, and monthly resolutions.

### AE documents

Design documents are provided with permission from the design team in a single.pdf file. The architectural floor plans, elevations, sections, and details provide massing context and enclosure thermal performance context. Schedules and notes accompany the scaled mechanical, electrical, and plumbing plans. The corresponding author verified the field conditions against the plans during the project’s original commissioning process. Finally, a schematic plan of the solar PV system was developed by the research team and included in the AE documents folder.

## Technical Validation

The authors recognize the importance of providing reliable measurements when reporting BPD. Measurement reliability is particularly important when data is sourced from a single, non-random case study since there are no other use cases to identify outliers. Table 4 summarizes the measurement instrumentation, associated standard(s), and accuracy of the study’s measurements.

**Table 4:** Data instrument, standard, and accuracy overview.

Category	Instrument	Standard	Accuracy
Energy Use	• SiteSage IOT Gateway	• UL 61010-1	+/− 1%
	• 150 A CT (n = 2)	• IEC 61869-2	
	• 50 A CT (n = 20)		
	• 20 A CT (n = 8)		
Energy Production	• Enphase Envoy Gateway Microinverter (n = 56)	• ANSI C12.20	+/− 0.5%
Building Air Leakage	• DG-700 Pressure and Flow Gauge (n = 2)	• ASTM E779	• DG-700: +/− 1%
	• Model 3 Blower Door Fan (n = 2)	• ASTM E1258-88	• Fan: +/− 3%
	• TECLOG3 data logging software		

The data presented in this descriptor required multiple instruments and represented multiple domains of BPD. A discrete experiment could not validate all data provided in this descriptor. During the measurement period, the research team periodically checked the energy use and production data via user interfaces for system status (e.g., on/off) and benchmarked the measured data against ASHRAE 90.1 and EnergyPlus simulations. Missing data were identified in the data cleaning process and further characterized in the dataset. Users of the dataset should recognize that the data is five years old and should account for changes over time in building technology, weather, and human-building interactions. The following section summarizes steps taken to ensure data reliability.

### Energy use

**Fig. 4:**
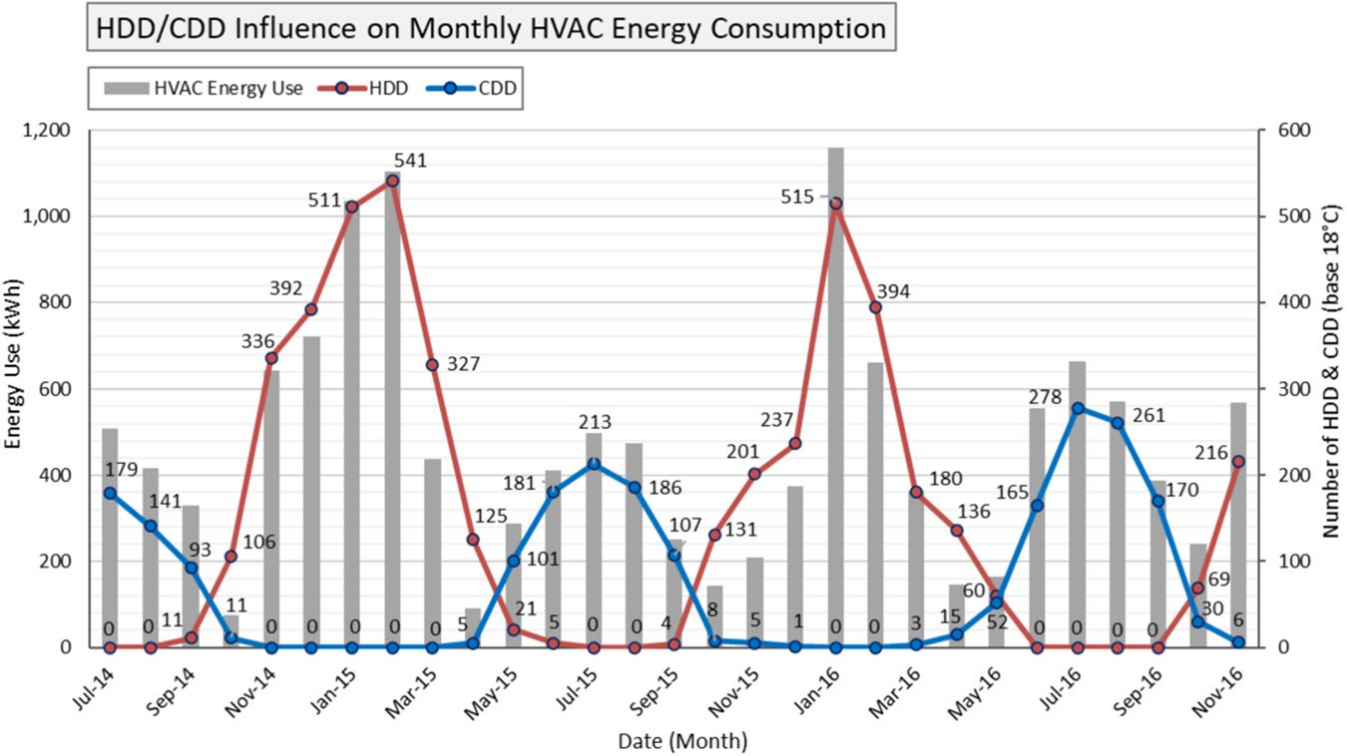
HDD/CDD (base 18 °C) influence on monthly HVAC consumption.

The longitudinal collection of energy use data is an important feature of this dataset. The thirty months of energy use data provided in this descriptor can be used to identify monthly and yearly outliers. The authors also benchmarked the whole building site energy use intensity (EUI) with energy standard performance. For example, the case building site EUI is 56.3 kWh/m^2^-yr, and the ASHRAE 90.1-2016 benchmark for small office buildings is 82.0 kWh/m^2^-yr^19^. Finally, the authors validated monthly heating and cooling consumption with monthly HDDs and CDDs data as energy demand over time (kWh) for heating and cooling correlates with HDDs and CDDs for the building location. Figure 4 demonstrates the influence of monthly HDD/CDD on monthly heating and cooling energy use.

### Energy production

The longitudinal collection of energy production data is an important feature of this dataset. The sixty-six months of energy production data provided in this descriptor can be used to identify monthly and yearly outliers. The provided weather data and solar radiation data can also be employed to correlate weather conditions with energy production outcomes.

### Building air leakage

**Fig. 5:**
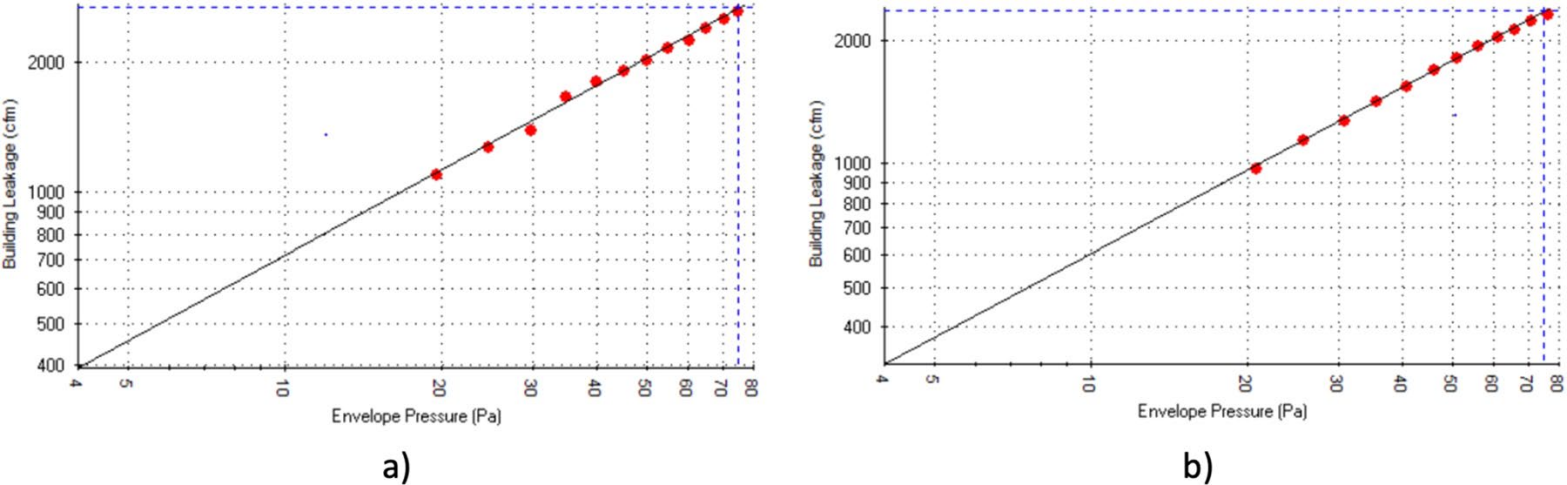
Building air tightness test results (**a**) pressurization and (**b**) depressurization.

Beyond the fan and gauge calibration requirements, the building air leakage test must follow, and results must meet several reliability steps. Specifically, the test requires flow rates to be sampled at a minimum of 10 test pressure points (between 20–75 Pa). Flows are averaged for at least 10 seconds per test point. Per the USACE test protocol and ASTM E779, test results must achieve a squared correlation coefficient (r^2^) ≥0.98. Both depressurization and pressurization tests resulted in a r^2^ = 0.99, thus passing the standard. Finally, during the test, TECLOG3 (e.g., building air leakage software) records the multi-point test results and generates curve fit to the Power Law Eq. (1) in compliance with ASTM E779.

1 $${\boldsymbol{Q}}={\boldsymbol{C}}\Delta {{\boldsymbol{P}}}^{n}$$

where *Q* is the infiltration/exfiltration rate, *C* is the air flow coefficient, Δ*P* is the pressure differences across the building enclosure, and *n* is the pressure exponent. The curve predicts infiltration rates from 0 Pa (0.0 inH_2_O) to 75 Pa (0.3 inH_2_O). See Fig. 5a for the pressurization curve and Fig. 5b for the depressurization curve.

## Data Availability

No code was used in the generation of this data. No code is required to access or analyze this dataset.

## References

[1] Roth J, Lim B, Jain RK, Grueneich D (2020). Examining the feasibility of using open data to benchmark building energy usage in cities: A data science and policy perspective. Energy Policy.

[2] Granderson J (2016). Accuracy of automated measurement and verification (M&V) techniques for energy savings in commercial buildings. Applied Energy.

[3] Huebner GM, Mahdavi A (2019). A structured open data collection on occupant behaviour in buildings. Sci. Data.

[4] Kriechbaumer T, Jacobsen H-A (2018). BLOND, a building-level office environment dataset of typical electrical appliances. Sci. Data.

[5] Kelly J, Knottenbelt W (2015). The UK-DALE dataset, domestic appliance-level electricity demand and whole-house demand from five UK homes. Sci. Data.

[6] Mahdavi A, Berger C, Tahmasebi F, Schuss M (2019). Monitored data on occupants’ presence and actions in an office building. Sci. Data.

[7] Ruhnau O, Hirth L, Praktiknjo A (2019). Time series of heat demand and heat pump efficiency for energy system modeling. Sci. Data.

[8] Schweiker M, Kleber M, Wagner A (2019). Long-term monitoring data from a naturally ventilated office building. Sci. Data.

[9] Miller C (2020). The Building Data Genome Project 2, energy meter data from the ASHRAE Great Energy Predictor III competition. Sci. Data.

[10] Rashid H, Singh P, Singh A (2019). I-BLEND, a campus-scale commercial and residential buildings electrical energy dataset. Sci. Data.

[11] Paige F, Agee P, Jazizadeh F (2019). flEECe, an energy use and occupant behavior dataset for net-zero energy affordable senior residential buildings. Sci. Data.

[12] Klemenjak C, Kovatsch C, Herold M, Elmenreich W (2020). A synthetic energy dataset for non-intrusive load monitoring in households. Scie. Data.

[13] Zhivov, A., Bailey, D. & Herron, D. USACE Air Leakage Test Protocol for Building Envelopes- Version 3 (2012).

[14] ASTM E779-10. *Standard Test Method for Determining Air Leakage Rate by Fan Pressurization*. http://www.astm.org/cgi-bin/resolver.cgi?E779-19 (2010).

[15] Agee P, Nikdel L (2021). Open Science Framework..

[16] Weather Data by Location. *EnergyPlus* https://www.energyplus.net/weather-location/north_and_central_america_wmo_region_4/USA/VA/USA_VA_Charlottesville-Albemarle.County.AP.724016_TMY3.

[17] Charlottesville, VA Weather History. *Weather Underground* https://www.wunderground.com/history/monthly/us/va/charlottesville/KCHO.

[18] NOAA NCEI. Integrated Surface Dataset (Global). *NOAA National Centers for Environmental Information* https://www.ncei.noaa.gov/access/metadata/landing-page/bin/iso?id=gov.noaa.ncdc:C00532 (2001).

[19] PNNL. *Energy Savings Analysis: ANSI/ASHRAE/IES Standard 90.1-2016*. https://www.energycodes.gov/sites/default/files/documents/02222018_Standard_90.1-2016_Determination_TSD.pdf (2017).

